# The Effect of Platelet-Rich Plasma on Bone Volume in Secondary Alveolar Bone Grafting in Alveolar Cleft Patients: A Systematic Review

**DOI:** 10.7759/cureus.46245

**Published:** 2023-09-30

**Authors:** Prem Vishva, Navaneethan R, Sruthi Harikrishnan

**Affiliations:** 1 Orthodontics and Dentofacial Orthopaedics, Saveetha Dental College and Hospitals, Saveetha Institute of Medical and Technical Sciences, Saveetha University, Chennai, IND

**Keywords:** secondary alveolar bone grafting, alveolar cleft, bone volume, bone grafting, platelet rich plasma

## Abstract

This systematic review aims to investigate the impact of platelet-rich plasma (PRP) in conjunction with bone grafting on bone volume outcomes in secondary alveolar bone grafting (SABG) procedures among alveolar cleft patients. An exhaustive search involving PubMed, Cochrane, and Google Scholar databases yielded 20 relevant titles, ultimately leading to the inclusion of four articles meeting all specified criteria. Based on the Cochrane risk of bias in systematic reviews (ROBIS) tool, the studies showed a high risk of bias. The primary outcome, bone volume assessment, was analyzed across these articles. While the Cochrane ROBIS tool deemed the included articles to have a high risk of bias, the comparison between PRP and Non-PRP groups did not reveal a significant difference in bone volume. Radiographic data illustrated an initial three-month period of bone resorption post-graft, regardless of PRP application, followed by a six-month phase of heightened bone density, particularly discernible in the PRP groups. To sum up, our findings indicate an absence of substantial bone density increase in cleft patients undergoing SABG with PRP augmentation. Nonetheless, there was a modest trend that suggests potential incremental bone density improvement with PRP usage, underscoring the need to conduct rigorously designed, randomized controlled trials (RCTs) with low bias to validate these observations.

## Introduction and background

Cleft lip and palate are the most common congenital defects affecting the orofacial area, and significant efforts have been made to characterize and repair these deformities. An alveolar bone graft has been normally deemed necessary for children with complete alveolar clefts during mixed dentition [1]. Secondary alveolar bone grafting (SABG) has been demonstrated to have a success rate of 90-95% in the repair of the cleft alveolus, with fresh autogenous bone being the most potent alternative for bone grafts since it contains living, osteogenic bone cells that are immunologically compatible [2]. SABG performed during the mixed dentition stage facilitates the proper eruption of canine teeth by creating a cancellous bone structure through the use of a bone graft [3,4]. Multiple sites can be utilized for the procurement of bone for grafting purposes. The use of autogenous bone grafts obtained from the ilium is largely acknowledged as the best and most dependable approach for performing SABG. The ilium is frequently employed because of its accessibility and capacity to facilitate substantial bone regrowth. Nevertheless, instances of unsuccessful outcomes have also been documented in relation to ilium-derived bone grafts in the cleft alveolus region, particularly in the vicinity of the nostrils and alveolar ridges [5-9].

Platelet-rich plasma (PRP), due to its osteoinductive qualities, has the ability to initiate and enhance the healing mechanisms, making the process involving it a very promising therapeutic approach for the treatment of bone, cartilage, and soft tissue abnormalities. The tissue healing process is enhanced by PRP thanks to the presence of crucial cytokines and growth factors (GFs). The use of PRP in conjunction with bone grafts for the treatment of infrabony defects has demonstrated efficacy in probing pocket depth, levels of attachment, and radio density [10-13]. The combination of PRP and autologous bone grafts during SABG has demonstrated improvements in initial bone growth, boosted bone density, and reduced bone resorption in individuals with alveolar cleft [14,15]. Prior research has employed radiographs as a means of quantifying bone turnover and bone density and demonstrated favorable outcomes associated with the application of PRP in conjunction with bone grafts.

In light of this, the primary objective of this systematic review was to evaluate how the utilization of PRP alongside secondary alveolar bone grafts influences the bone volume within maxillary alveolar clefts on radiographic assessment.

## Review

Study protocol and research question

In conducting this review, we followed the Preferred Reporting Items for Systematic Reviews and Meta-Analyses (PRISMA) checklist. The research question addressed in this systematic review was as follows: "Does platelet-rich plasma have an effect on bone volume change in alveolar clefts after bone grafting?''. The population, intervention, control, and outcome (PICO) elements of the systematic review are shown in Table 1.

**Table 1:** PICO elements of the systematic review PICO: population, intervention, control, and outcome

Elements	PICO
Population	Alveolar cleft surgery patients
Intervention	Platelet-rich plasma on bone grafting
Comparison	Bone grafts without platelet-rich plasma
Outcome	Bone volume evaluation

Search method for the identification of studies and sources

To identify the studies to be included for detailed evaluation in this systematic review, the following databases were searched: PubMed Central, the Cochrane Central Register of Clinical Trials, Google Scholar, Trip, and Science Direct. We searched for studies employing cross-sectional design published till February 2023. The search terms used were as follows: "randomized controlled trial," "controlled clinical trial," "clinical trial," "platelet-rich plasma," "autologous bone graft with PRP," "PRP on bone grafting," "bone grafting," and "secondary alveolar bone grafting." Further cross-references were manually searched for.

The search initially yielded a total of 3338 studies. Two authors (PVN, NR) independently screened titles to identify relevant studies based on predetermined inclusion and exclusion criteria. Conflicts concerning the selection of studies were settled by discussions. Twenty articles were identified based on exclusion by reading titles and removing duplicates. Abstracts of selected articles were further reviewed independently and 13 more articles were excluded. Three more articles were removed after reading the full text. Ultimately, four articles were selected based on eligibility criteria for the final analysis. 

Inclusion Criteria

Clinical trials evaluating the bone volume on alveolar cleft surgery with PRP in bone graft placement; randomized controlled trials (RCTs) that evaluate the bone volume after bone grafting with PRP in alveolar cleft surgery; studies published in the English language

Exclusion Criteria

Non-English studies; animal studies; in vitro studies; studies involving platelet-rich fibrin (PRF)

**Figure 1:**
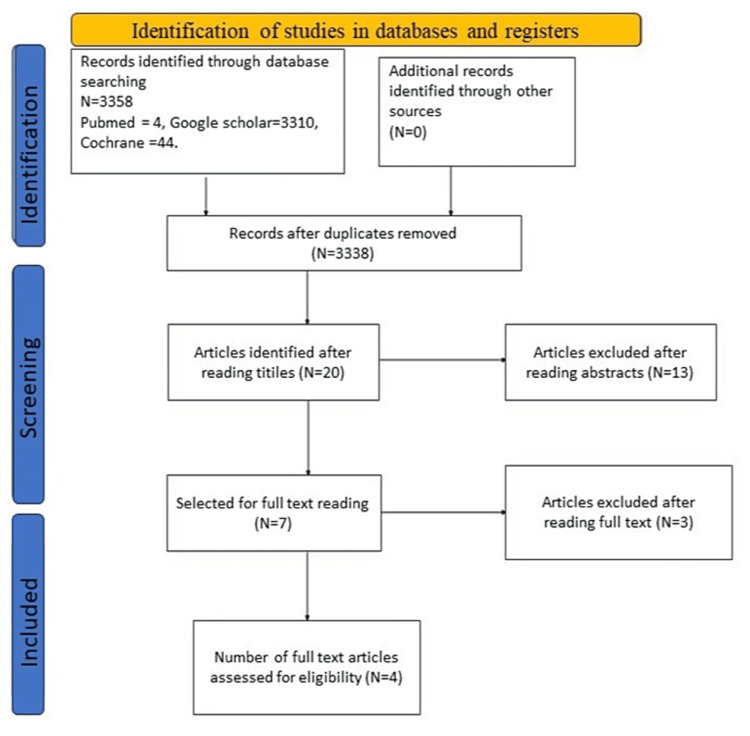
PRISMA flow diagram depicting the selection of included studies PRISMA: Preferred Reporting Items for Systematic Reviews and Meta-Analyses

The reference list of studies with full text was examined in order to find out if more studies could be incorporated. The selection of article titles pertinent to the evaluation was determined through a process of discussion. The abstracts of the two chosen articles were examined. The divergence of viewpoints about the incorporation of a particular study was successfully resolved through deliberation, resulting in the exclusion of both articles subsequent to a thorough examination of their abstracts. The authors independently applied the PRISMA standards to assess the quality of the research. The authors also conducted an independent assessment of the risk of bias for each study, and any conflicts in this regard were resolved by discussion. Figure 1 depicts the PRISMA flow diagram detailing the study selection process.

Assessment of the Quality of Included Studies

The evaluation of study quality adhered to the guidelines detailed in the Cochrane Handbook of Systematic Review. The assessment encompassed the following key parameters: random sequence generation and allocation concealment to address selection bias; blinding of participants and personnel and outcome assessment to mitigate performance and detection bias; scrutiny of incomplete outcome data to ensure data integrity; examination of baseline balance to prevent reporting bias; and ensuring adequate reliability. These comprehensive criteria were employed to rigorously appraise the studies included in this analysis.

Risk of Bias

All parameters were evaluated to determine their classification as high risk, low risk, or unclear risk. The final risk of bias of an individual study was determined as low risk if the study showed low risk for all the individual parameters. If there was a high or uncertain level of risk associated with one or two criteria, the included study was categorized as having a moderate level of risk. If the included study exhibited high risk or uncertain risk with regard to more than two indicators, it might be inferred that the study had a significant risk of bias.

Results

A total of 20 titles were found using the search method. Following the screening process, 13 papers were disqualified as their titles and abstracts did not match the criteria for inclusion. The remaining seven articles were read in their entirety, of which three were excluded as they did not evaluate PRP. Finally, after careful analysis, four papers that met our criteria for inclusion were included in this review. The salient attributes of the included studies are presented in Table 2.

**Table 2:** Characteristics of included studies CBCT: cone-beam computed tomography; CT: computed tomography; Al-Eq: aluminum-equivalence; PRP: platelet-rich plasma; ANOVA: analysis of variance; RCT: randomized controlled trial

Author and year of study	Study design	Sample size	Groups (intervention and control)	Outcomes assessed	Statistical methods used	Tools used for assessment
Bezerra et al. [16]	Clinical trial, pilot study, prospective	20 cleft patients	Intervention group: i - autologous bone graft, ii - bone graft + PRP	Radiographic assessment of the area and volume - CBCT	Paired t-test, Student's t-test	Area/volume tool, both on the preoperative and postoperative CBCT scans for each patient
Gupta et al. [17]	RCT	20 patients	Group 1: autogenous bone graft with PRP. Group: without PRP	Bone density of the grafted bone was assessed with DentaScan, using pixel tools image analyzer software	Repeated measures ANOVA	Study model measurements: bone density of the grafted bone was assessed with DentaScan, using pixel tools image analyzer software, at regular postoperative follow-up at 1, 3, and 6 months
Lee et al. [18]	Clinical control trial	60 patients	Group A: 30 patients with grafted autogenous bone and PRP (PRP group). Group B: 30 patients with grafted autogenous bone alone (non-PRP group) were enrolled	Bone density was quantitatively assessed as an Al-Eq value	One-way ANOVA, Pearson correlation	The density and resorption of grafted bone were evaluated at 1 week, and 1, 3, 6, and 12 months postoperatively
Sakio et al. [19]	Clinical control trial	29 patients	Group A (control group ): 6 patients received iliac cancellous bone and marrow grafts without PRP. Group B: 23 other patients were included in the PRP group and received grafts with PRP	Bone volume evaluation with computer-aided engineering with multidetector row CT	Wilcoxon matched-pairs rank test or Pearson coefficient of correlation, as appropriate	Evaluation was done with 320-multidetector row CT before and at 1 month and 1 year post operation

The studies incorporated in the review assessed the process of bone development in the cleft region following SABG across several timeframes: at one month, three months, six months, and one year. Twenty patients with clefts were included in the study by Bezerra et al., 10 boys and 10 girls, with a mean age of 15.6 years in group A and 14.5 years in group B. The mean size of the cleft defect was 152 ±345.81 mm^2^ in group A and 274.39 mm^2^ in group B prior to surgery. Postoperatively, the mean area of the cleft dropped to 132.72 in group B and 265.08 in group A. The mean volumes were 277.87 mm^3^ and 153 ±374.88 mm^3^ in groups B and A, respectively. Patients in group B had their mean cleft volume decreased to 96.19, whereas patients in group A had their mean cleft volume decreased to 254.12 [16]. In the study by Sakio et al., non-syndromic individuals with unilateral clefts were treated with alveolar bone grafting with or without PRP; the one-month postoperative bone volume was 1.00 ±0.53 cm^3^ and 1.29 ±0.33 cm^3^, respectively. The bone volume one year after surgery, with or without PRP, was 0.55 ±0.44 and 0.59 ±0.28 cm^3^, respectively. There was no discernible difference between the mean resorption ratios: 49.9 ±17.2% vs. 44.9 ±14.4% [19].

Gupta et al. studied 20 individuals with unilateral or bilateral cleft lip and palate and alveolar clefts, whose ages ranged from eight to 30 years. In group A, 90% of patients experienced primary healing, while the remaining 10% experienced secondary healing within 180 days; 30% of patients in group B experienced secondary healing. At six months postoperatively, the bone density of bone grafts with extra PRP was greater than it was for grafts without PRP. There was a statistically significant increase in density detected. Nevertheless, at three months, the PRP group's mean bone density was 1.04 times greater than that of the non-PRP group, and 1.2 times greater after six months, according to the DentaScan image analyzer software [17]. The study by Lee et al. involved 60 individuals who underwent secondary bone grafts in the alveolar cleft. In all cohorts, the overall rate of aluminum-equivalence(Al-Eq) in the transplanted bone exhibited a gradual decrease until the three-month mark, followed by a subsequent increase until the 12-month mark. Each dental X-ray film's radiological density of grafted bone was calculated as an Al-Eq value, proportional to Al-Eq values of the bone mineral content. At three months, the average Al-Eq rate in the PRP group was 79.3%, and at 12 months, it was 93.5%. The non-PRP group had 85.2% at three months and 90.0% at twelve months. The Al-Eq rate was considerably lower in the PRP group than in the non-PRP group at three months [18].

The mean values and statistical significance were used to conduct a comprehensive assessment of the observed variables in the study. The comprehensive results of the included study details have been systematically organized for easy reference and efficient data analysis, as detailed in Table 3.

**Table 3:** Summary of the findings of included studies PRP: platelet-rich plasma

S. no	Study	Results		
		Postoperative bone volume		
1.	Lee et al., 2009 [18]	12 months postoperatively - PRP group: 0.94 to 2.54 cm^3^; non-PRP group: 0.59 to 2.16 cm^3^		
2.	Gupta et al., 2013 [17]	6 months postoperatively - PRP group: 1028.00 ±11.30 HU; non-PRP group: 859.50 ±27.73 HU		
3.	Sakio et al., 2017 [19]	1 month postoperatively - PRP group: 1.00 ±0.53 cm^3^; non-PRP group: 1.29 ±0.33 cm^3^. 1 year postoperatively - PRP group: 0.55 ±0.44 cm^3^; non-PRP group: 0.59 ±0.28 cm^3^		
4.	Bezerra et al., 2019 [16]	Groups	Preop	1-year postop
		Autologous bone graft + PRP		
		Area, mm^2^	345.81 (143.60)	265.08 (180.96)
		Volume, mm^3^	374.88 (194.65)	254.12 (195.78)
		Bio-Oss + PRP		
		Area, mm^2^	274.39 (75.51)	132.72 (86.81)
		Volume, mm^3^	277.87 (107.01)	96.19 (76.60)

**Figure 2:**
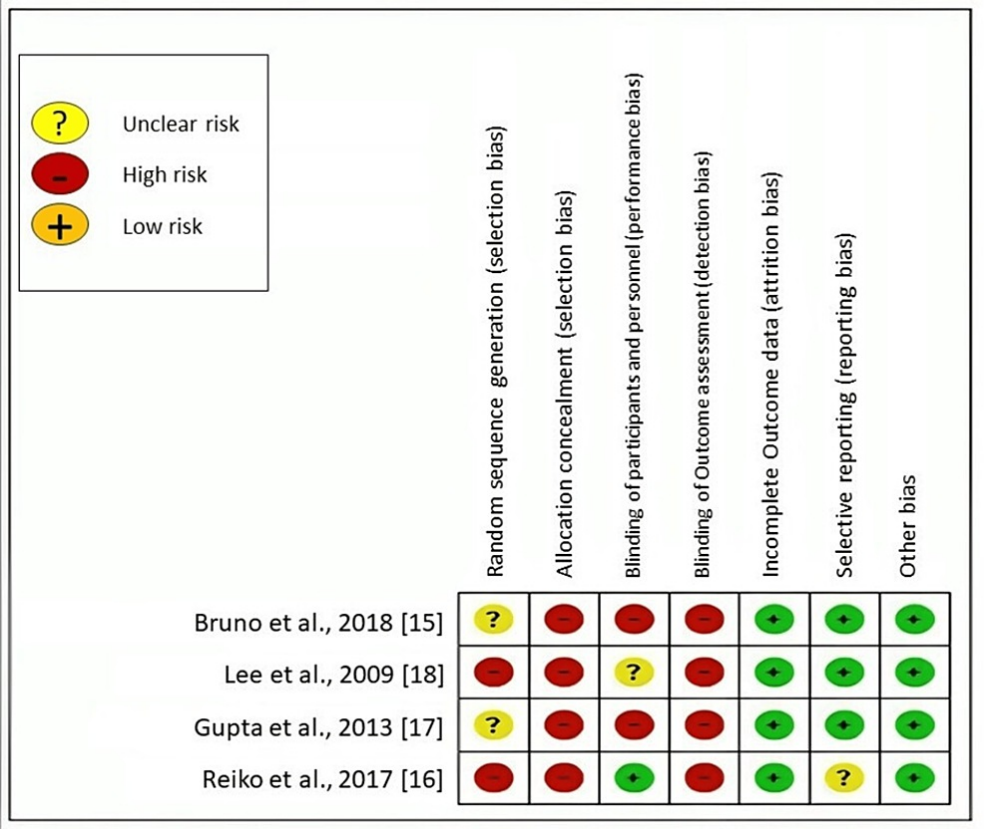
Research quality of the included studies as assessed by the Cochrane risk of bias in systematic reviews (ROBIS) tool

The Cochrane risk of bias in systematic reviews (ROBIS) tool was utilized to evaluate the potential for bias in each study. The results of the risk of bias assessment for the included studies are summarized in Figure 2. The majority of studies were determined to possess a significant risk of bias. Overall, the majority of the studies analyzed were found to possess an indeterminate level of bias in the areas of allocation concealment, random sequence generation, participant blinding, and outcome assessment blinding. However, the majority of the studies showed a low risk of bias in areas such as insufficient outcome data, selective reporting, and other potential sources of bias. In the course of these trials, it was not feasible to achieve blinding for both the clinician and the patient. In some of the studies included in the analysis, the allocation concealment strategy led to a greater number of participants in the experimental group compared to the control group. This discrepancy in participant allocation introduces a significant risk of bias in the included research.

Discussion 

All studies consistently demonstrated that PRP application enhanced bone formation. There has been no further systematic review/meta-analysis of the current data on the outcomes of PRP combined with SABG in cleft palate repair. This review involves four RCTs focusing on the effectiveness and safety of traditional secondary bone grafting with autogenous bone in combination with other bone regenerative growth factors like PRP in treating an alveolar cleft defect in children with cleft lip and/or palate. The amount of bone formation was found to be high in all four studies. The evidence was assessed as poor quality, requiring the physician to use caution when making pertinent recommendations. There were no documented adverse responses in any of the investigations. Unfortunately, in the vast majority of instances, conclusive proof is still lacking.

The method of evaluation also differed between studies. In the study by Lee et al. [18], a two-dimensional evaluation was performed to examine the Al-Eq scores at various time points: one week, and one, three, six, and 12 months postoperatively. In the study conducted by Gupta et al. [17], the density of the transplanted bone was assessed using DentaScan and pixel tools image analysis software at three separate time points: one, three, and six months. The study by Saiko et al. [19] involved the use of CT scans before the procedure, as well as at one and 12 months postoperatively. The standardization of patient positioning involves aligning the maxillary alveolar crest parallel to the plane of the scan. Bruno et al. employed cone-beam CT scans of the maxilla, which were subsequently transformed into three-dimensional (3D) models, in order to examine the magnitude and volume of the cleft defect both prior to and one year following the surgical intervention. According to the findings of Lee et al. [18], PRP has the potential to improve the process of bone remodeling during the initial stages of treatment.

The PRP group exhibited a higher rate of Al-Eq values at 12 months (97.8%), indicating enhanced bone remodeling. However, the long-term effectiveness of PRP in preventing bone resorption following a subsequent bone transplant in the alveolar cleft remains uncertain when combined with grafting techniques. Based on the findings of Gupta et al. [17], the combination of autologous bone chips obtained from the iliac crest with PRP has been observed to promote bone formation in alveolar clefts. This combination has demonstrated several advantages, including expedited bone formation, increased bone density, reduced infection rates, and minimal postoperative discomfort. Specifically, at the three-month mark, the PRP group exhibited a significantly lower level of discomfort compared to the non-PRP group, while at the six-month mark, the PRP group experienced 1.2 times more discomfort.

Evaluation by Sakio et al. [19] noted no discernible difference between the mean resorption ratios of the PRP group and the non-PRP group, and, after a year, the postoperative bone resorption following autogenous cancellous bone grafts did not seem to be significantly reduced by PRP. Bezerra et al.'s [16] pilot study showed a positive outcome demonstrating that such PRP combined with bovine graft (Bio-Oss) is a viable choice for the repair of alveolar clefts where results were similar to autologous bone grafts when compared to PRP with bovine graft, and hence it can serve as a feasible alternative when autologous bone cannot be acquired.

Mechanical stress was designed to force nearby teeth into the grafted bone either through spontaneous eruption or orthodontic force (orthodontics cases). Apart from bone regeneration rate, bone resorption rate was also estimated in a few included studies [19]. When comparing the resorption rates of grafted bone in the groups treated with PRP and those without PRP, it was observed that the bone bridge subjected to mechanical stress exhibited a reduced resorption rate compared to the one subjected to non-mechanical stress. This endorsed the findings of earlier studies that indicated persistent mechanical stress in the alveolar cleft would stop bone resorption. When it comes to the effect of PRP on bone resorption, non-orthodontic cases (36.4%) had a higher rate of resorption than orthodontic cases (32.3%), which suggests that PRP did not inhibit bone resorption in the event of disuse atrophy in the absence of prolonged mechanical stress following SABG in palatal cleft [20,21]. Several other studies have observed enhanced bone density and graft integration in patients who underwent SABG along with PRP, contrasting with the outcomes of graft-only procedures. PRP-augmented grafting led to expedited bone healing and a lower incidence of graft-related complications [22,23].

This study has many strengths, one of which is its adherence to a well-established methodology. The technique utilized to obtain data via both manual and electronic sources up to February 2023 was extensive, with no predetermined constraints on terminology, date of publication, or publishing status. Additionally, the screening, verification of eligibility, abstraction of information, risk of bias assessment, and assessment of the quality of evidence were exhaustively undertaken to minimize potential biases. Disagreements were settled by discussion until a consensus was achieved.

The evidence from the outcomes reported is inconclusive, with some aspects suggesting an improved outcome, such as those linked to volumetric assessments from CT, while others suggesting no difference in the outcome, such as those pertaining to defects in height or width reduction. The PRP with bone grafts could be utilized as an alternative to autologous bone graft without PRP, but further research is required before it could be definitively recommended. If the human iliac bone is to be used as a graft material, the addition of the artificial material, as investigated in one trial, does not appear to affect the outcome when assessed by radiographic parameters. The use of adjuncts to harvested autogenic bone also requires further research to clarify the benefits to patients and clinicians. This systematic review has not been able to achieve all the objectives described in the protocol. Furthermore, no RCTs studying the optimal timing and the sequence of orthodontic treatment with secondary bone grafting surgery were found in our search. The existing body of research, which is currently limited in scope and may be subject to a significant risk of bias, does not provide definitive evidence about the enhancement of therapeutic results. Further trials are necessary in order to establish conclusive findings and provide recommendations pertaining to the use of SABG in pediatric patients with cleft lip and/or palate.

## Conclusions

Based on our findings, bone regeneration was on the higher side in the first three months in patients who underwent autologous bone grafting without PRP, whereas bone formation was more pronounced in the following six months in those who underwent bone grafting with PRP. The results of articles included in this systematic review were statistically insignificant in terms of bone volume. Consequently, further, more rigorously designed RCTs are required to examine the efficacy of PRP grafting for bone regeneration.
